# Biochemical and Physiological Profiles of *Nakaseomyces glabratus* Isolates from Bulgarian Clinical Samples

**DOI:** 10.3390/life15060889

**Published:** 2025-05-30

**Authors:** Nadja Radchenkova, Penka Stefanova, Dilnora Gouliamova

**Affiliations:** 1 Departament of General Microbiology, The Stephan Angeloff Institute of Microbiology, Bulgarian Academy of Sciences, G. Bonchev 26, 1113 Sofia, Bulgaria; 2 Saint George Hospital, Medical University, Vasil Aprilov Blvd 15, 4002 Plovdiv, Bulgaria; pepi_stef@hotmail.com

**Keywords:** *Nakaseomyces glabratus*, opportunistic pathogens, enzymatic activities, antifungal drug resistance

## Abstract

*Nakaseomyces glabratus* is an opportunistic fungal pathogen that primarily affects immunocompromised individuals. Unlike other *Candida* species, *N. glabratus* exhibits nondimorphic blastoconidial morphology and a haploid genome. It is a leading cause of both superficial (oral, esophageal, vaginal, or urinary) and systemic candidiasis. In this study, we evaluated 47 clinical isolates from Central Bulgaria (Plovdiv) and 1 wild strain isolated from the gut of the beetle Oxythyrea funesta (Coleoptera: Cetoniinae) collected in Sofia, Bulgaria. Growth was observed across a pH range of 3 to 9. The strains were assessed for the production of lipases, esterases, and proteases—enzymes associated with pathogenicity—and their relationship to virulence. Biofilm formation and exopolysaccharide production were also measured, with all strains showing similar profiles. No competitive inhibition of *N. glabratus* was observed against *C. parapsilosis*. All isolates exhibited resistance to micafungin, while resistance to both micafungin and anidulafungin was observed in 21 isolates (44%). These findings provide insight into the biochemical characteristics of *N. glabratus* populations from Southeast Europe, contributing to a better understanding of strain behavior under controlled laboratory conditions and addressing the gap in data on this species in the region.

## 1. Introduction

*Candida* spp. are part of the natural microbiota in healthy individuals. However, under conditions of compromised host immune status, *Candida* isolates can cause serious health problems [1]. Recent data from the European Confederation of Medical Mycology (ECMM) revealed a decrease in the prevalence of *C. albicans* (56.4%) and an increase in *C. glabrata* (13.6%). Regionally, the highest proportion of C. albicans was observed in Austria (77%), *C. parapsilosis* in Italy and Turkey (24–26%), *and C. glabrata* in the Czech Republic, France, and the UK (25–33%) [2]. The paucity of data from Bulgaria, combined with the reported increase in *N. glabrata* infections across Europe, underscores the growing relevance of this pathogen. *Nakaceomyces glabratus* (formerly known as *C. glabrata*) is characterized by metabolic flexibility [3]. While it cannot efficiently utilize a broad range of carbon sources, this limitation does not limit its proliferation in diverse environments. This metabolic adaptability is mediated by a regulatory network that facilitates the organism’s adjustment to nutrient availability and other environmental fluctuations. Such versatility contributes to its persistence in the human host and its potential to cause infection. *N. glabratus* can colonize mucosal surfaces of the mouth, esophagus, intestines, and vagina, yet information regarding host–pathogen interactions and immune defense mechanisms remains insufficient. It is hypothesized that host immune mechanisms regulate *N. glabratus* by suppressing the expression of its virulence properties, thereby preventing infection [4,5]. Phylogenetically, *N. glabratus* is distantly related to other *Candida* species and is more closely related to *Saccharomyces cerevisiae* [6]. This evolutionary divergence is thought to have endowed *N. glabratus* with distinct morphogenetic and pathogenic traits, aiding in its ability to evade the host immune system. Although *N. glabratus* is considered predominantly asexual, genomic analyses of clinical isolates have revealed substantial genetic diversity, particularly in chromosome structure and sequence [7,8]. Understanding the genetic diversity of *N. glabratus* isolates, both clinically and biologically, is crucial for addressing key questions related to its epidemiology and for developing effective infection control strategies by identifying its transmission pathways [9]. It also contributes to understanding the evolution of drug resistance in genetically related isolates during infection [10].

The present study examines pH as a critical factor influencing the growth and survival of yeasts. *N. glabratus* has been isolated from both environmental sources and clinical samples. The pH within these niches can vary significantly, prompting an investigation into the organism’s response to extreme pH conditions, particularly acidic and alkaline environments [11]. Furthermore, pH plays a role in the expression of virulence factors [12], including enzymes that facilitate evasion of host immune responses [13]. Extracellular lipases are proposed to have several roles in pathogenicity, including (1) the digestion of lipids for nutrient acquisition, (2) adhesion to host cells and tissues, (3) synergistic interactions with other enzymes, (4) the initiation of inflammatory responses through immune cell modulation, and (5) self-defense mechanisms that involve the degradation of competing microbiota. Extracellular lipases have been identified as potential virulence factors in a variety of bacterial pathogens, such as *Staphylococcus aureus* [14], *Staphylococcus epidermidis* [15], *Propionibacterium acnes* [16], and *Pseudomonas aeruginosa* [17], as well as in yeast pathogens. Numerous yeast species secrete lipolytic enzymes, including lipases and esterases [18,19]. These enzymes can damage host cell membranes, leading to cell lysis and facilitating yeast invasion [20]. Species such as *Kluyveromyces marxianas*, *Pichia kudriavzevii*, *N. glabratus*, and *M. guilliermondii* have been shown to produce proteolytic enzymes with relatively low activity in vitro [21], highlighting the importance of studying these organisms. These species also express surface proteins, known as adhesins, which allow cells to adhere to and colonize endothelial and epithelial tissues, including medical devices. Once colonization occurs, *N. glabratus* forms a multicellular biofilm structure composed of adhered cells embedded in a self-secreted polymeric matrix [22]. Biofilm formation plays a critical role in the pathogenicity of fungal infections. *Candida yeast* species, particularly *C. albicans*, *C. parapsilosis*, and *C. tropicalis*, as well as *N. glabratus* are associated with biofilm-related infections [23,24]. The cell wall of *N. glabratus* consists of a complex matrix-containing proteins, polysaccharides, and lipids, which promote adhesion and offer protection against host immune responses and antifungal agents. *N. glabratus* biofilms form multilayered structures of yeast cells. Biofilm formation is influenced by factors such as the choice of biomaterial, medium composition, and carbohydrate source and concentration [25,26]. *Candida* yeasts biofilms and *N. glabratus* are particularly resistant to azoles and amphotericin B but are susceptible to echinocandins [27]. *Nakaseomyces glabratus* is a significant opportunistic pathogen responsible for life-threatening infections and capable of biofilm formation on medical devices. Limited information exists regarding the composition of the *N. glabratus* biofilm matrix, though it is known to contain proteins and carbohydrates, including exopolysaccharides such as β1,3-glucans [28,29,30].

In addition, in our study, we investigated in vitro interaction between *N. glabratus* and *C. parapsilosis*. Although both species are clinically significant, little is known about their potential competitive or cooperative behaviors when cultured together. Each yeast pathogen species holds unique characteristics regarding invasive potential, virulence, and antifungal susceptibility pattern [31].

It has been noted that *N. glabratus* has shown an increase in resistance to echinocandin over the past decade, ranging between 1.5% and 12%, with increasing frequency and multidrug resistance [32,33]. Unfortunately, this alarming trend is confirmed by the current study. The aim of this work was to study physiological profiles of *N. glabratus* in laboratory conditions in the absence of a competitive host environment and virulence factors in different isolates as well as to determine the sensitivity to antifungal drugs. Our results demonstrate the profile of this opportunistic pathogen from an unexplored region of Eastern Europe, Bulgaria.

## 2. Materials and Methods

### 2.1. Isolation of Yeasts

Environmental isolate D77_3 obtained from the gut of beetle and 47 isolates obtained from clinical samples from Saint George Hospital were inoculated in Microbiology Department of Medical University on Yeast Malt Agar (YMA) medium containing 0.3% yeast extract (Fisher Scientific, Waltham, MA, USA) 0.3%, malt extract (Oxoid, Cheshire, UK), 0.5% peptone (Merck, Darmstadt, Germany), and 2% glucose (Merck, Darmstadt, Germany) medium. To ensure purity, the yeast isolates were subcultured three times.

Genomic DNA was extracted from the yeast strains using the Bacterial & Yeast Genomic DNA Purification Kit (Gene MATRIX, Gdańsk, Poland) according to the manufacturer’s protocol.

All the strains were maintained in YPGA medium (0.3% yeast extract, 1% glucose, 0.5% peptone, 0.3% malt extract and 2% agar) at 4 °C.

### 2.2. Yeast Identification

Physiological characterization of the isolates was performed according to established methods [34].

### 2.3. Genomic DNA Extraction and Amplification and Sequencing of ITS1+2 Region

The primer sets V9G and LS266 were used for the amplification of the ITS-LSU rDNA region [35]. A cell suspension of one loopful of cells in 50 μL of sterile water was heated at 95 °C for 5 min, and 2 μL of the supernatant was used as a template in 25 μL PCR mixture containing 7.5 μL of PCR master mix (Fermentas, Darmstadt, Germany) and 0.5 μM of each primer. The amplified products were purified using a QIAquick purification kit (Qia Gen, Germantown, MD, USA) according to the manufacturer’s instructions. Direct sequencing of the ITS was performed using primers ITS1/ITS4 [36], with a Taq Dye Deoxy terminator cycle sequencing kit (Applied Biosystems, Foster City, CA, USA) by Macrogen Inc. (Seoul, Republic of Korea), according to the manufacturer’s protocol. Purified sequencing reaction mixtures were separated with a 3730XL automated DNA analyzer (Applied Biosystems, Foster City, Foster City, CA, USA). The program BLAST version 2.16.0 from NCBI (https://www.ncbi.nlm.nih.gov, accessed on 10 May 2025) was used to compare the sequences to known ITS sequences for the assignment of the closest related taxa. All the ITS sequences from this study were submitted to the GenBank database under the accession numbers for clinical isolates ON016539–ON016585; isolate D77_3-JQ026352.1.

### 2.4. pH Measurement

Strains were tested in basal YPG liquid medium (2% yeast extract, 10% peptone, 2% glucose), sterilized by autoclaving for 15 min at 110 °C. After sterilization, the pH was adjusted and tested across a wide range, with increments of 0.5. Overnight cultures of *N. glabratus* were adjusted to a turbidity equivalent to the 1.0 McFarland standard (Merck, Darmstadt, Germany) using a 0.9% NaCl solution. McFarland solution was then added to test tubes to achieve a final yeast inoculum concentration of 1%. The samples were cultured at 25 °C for 5 days. The population density of yeasts at different pH levels was assessed by visually comparing the turbidity of the cultures with a McFarland card.

The pH values of the isolates were measured using a glass electrode connected to a portable pH meter (pH 211, HANNA Instruments, Woonsocket, RI, USA) at 25 °C. The pH meter has a measurement range of pH 1–14, a resolution of 0.01 pH units, and an accuracy of ±0.01 pH units. Prior to measurement, the pH meter was calibrated using three standard solutions (pH 4.00, pH 7.00, and pH 10.00).

### 2.5. Enzymatic Activities

#### 2.5.1. Lipase Activity

Strains were tested on tributyrin agar medium composed of 0.5% peptone, 0.3% yeast extract, 1% tributyrin, 1.5% agar, and pH 6.0. The medium was sterilized by autoclaving for 15 min at 121 °C. The formation of a clear halo around the colony in an otherwise opaque medium demonstrated lipase activity [37]. The plates were incubated at 27 °C and observed for 5 days.

#### 2.5.2. Esterase Activity

Tween 80 (Merck, Darmstadt, Germany) was separately added to modified Sabouraud liquid broth (Oxoid, Cheshire, UK) to a final concentration of 1%. One percent agarose (Merck, Darmstadt, Germany) was also added as a solidifying agent. The mixture was autoclaved at 121 °C for 15 min. Overnight cultures were transferred to the substrate test medium via swab inoculation. The plates were incubated at 27 °C and observed for 5 and 7 days. Esterase activity was detected by the formation of zones of precipitation around the colonies, observed under transmitted light. These precipitates are formed by the hydrolysis of Tween 80, where the fatty acids released combine with calcium ions in the medium to form insoluble calcium salts [18,19].

#### 2.5.3. Protease Activity

The medium comprised of 0.5% peptone, 0.5% yeast extract, 0.5% glucose, and 2% casein, pH 6.5. The medium was sterilized by autoclaving for 15 min at 110 °C. The diameter of the colony and precipitation zone was measured according to protocol described previously [38]. The plates were incubated at 27 °C and observed for 5 days.

#### 2.5.4. Assimilation of Soluble Starch

The medium contained 0.67% YNB (yeast nitrogen base, HiMedia Laboratories Pvt. Ltd., Haiderabad, India), 0.2% soluble starch, and 2.0% agar, pH 6.0, sterilized by autoclaving for 15 min at 110 °C. The growth of strains in the specified medium indicated assimilation of soluble starch.

#### 2.5.5. Arbutin Test

Yeast strains were screened on Petri plates with medium composed of 10% yeast extract, 2% agar, 0.5% arbutin, and 40 drops/100 mL of a 1% ferric ammonium citrate solution, sterilized by autoclaving for 15 min at 121 °C. Each plate was inoculated with yeast isolate and incubated at 25 °C for 10 days. *Saccharomyces cerevisiae* ATCC 4098 was used as a negative control [39].

### 2.6. Biofilm Formation and EPS Sythesis–Crystal Violet Assay

#### 2.6.1. Crystal Violet Assay

The Crystal Violet colorimetric assay, which measures the uptake of the dye Crystal Violet was applied to assess biofilm formation. Reagents used were 0.4% aqueous Crystal Violet solution (Merck, Darmstadt, Germany), 95% Ethanol, and Phosphate-Buffered Saline (PBS) ph-7.4 (Merck, Darmstadt, Germany). After 24 h of biofilm growth, the medium was aspirated from the wells. The wells were washed twice with 200 μL of PBS. PBS was aspirated, and the wells were left to dry at room temperature for 45 min. The dried wells were stained with 110 μL of 0.4% Crystal Violet solution for 45 min. The wells were rinsed four times with 200 μL of distilled water to remove excess dye. The samples were then destained by adding 200 μL of 95% ethanol during 45 min. Ethanol (95%) was used as a control. The absorbance of the samples was measured at 595 nm using iMark™ Microplate Reader (Bio-Rad, Hercules, CA, USA) [40].

#### 2.6.2. Exoplysaccharide (EPS) Synthesys

The Minimum Salt Medium (MSM) was used for exopolysaccharide (EPS) synthesis. The composition of the medium, sterilized by autoclaving at 110 °C for 15 min, is as follows: 4.0% sucrose, 0.1% yeast extract (YE), 0.25% (NH₄)₂SO₄, 0.05% MgSO₄, 0.1% KH₂PO₄, 0.01% NaCl, and 0.01% CaCl₂, pH-4.3 [41].

#### 2.6.3. Isolation of EPS

Cells were harvested by centrifugation at 4000× *g* for 20 min, and the supernatant was used for EPS recovery. The supernatant was treated with an equal volume of cold absolute ethanol, added dropwise with stirring in an ice bath. The mixture was left at −18 °C overnight for complete EPS precipitation, then centrifuged at 13,000× *g* for 30 min. Carbohydrate analysis was performed using the phenol/sulfuric acid method [42], with glucose as the standard.

### 2.7. Antifungal Activity Against C. parapsilosis ATCC 22019

Strains were tested on YPG medium (2% yeast extract, 1% peptone, 2% glucose), which was sterilized by autoclaving at 110 °C for 15 min. Overnight cultures of *N*. *glabratus* and *C. parapsilosis* were adjusted to a turbidity equivalent to the 1.0 McFarland standard using a 0.9% NaCl solution. The surface of the agar plates was uniformly inoculated with 100 μL of *C. parapsilosis* suspension using a sterile swab. Wells were then made in the agar, each filled with 200 μL of *N. glabratus* culture supernatant, which was obtained by centrifuging the overnight culture at 10,000× *g* for 10 min to remove the cells. Plates were incubated at 37 °C for five days, and antifungal activity was assessed by the appearance of clear inhibition zones around the wells.

#### 2.7.1. Culturing of Yeast Isolates

*Nakaseomyces glabratus* isolates were cultured on Sabouraud Dextrose Agar (SDA) medium containing 4% dextrose, 1% peptone, and 1.5% agar, pH 5.6 (Oxoid, Cheshire, UK), including the reference strains *Candida parapsilosis* ATCC22019 and *Pichia kudriavzevii* ATCC6258. Plates were incubated at 37 °C for 3 days.

#### 2.7.2. Preparation of Inoculum

Yeast colonies grown on Yeast Malt (YM) agar were suspended in 0.85% sterile saline solution and adjusted to a turbidity equivalent to a 0.5 McFarland standard. A sterile cotton swab was soaked in the inoculum suspension, and the surface of Mueller–Hinton Agar (MHA) plates supplemented with 0.5 µg/mL Methylene Blue Dye (HiMedia, Mumbai, India, Laboratories Pvt. Ltd., Haiderabad, India) was evenly streaked in three directions to ensure uniform lawn growth. The MHA medium was composed of 0.2% beef extract, 1.75% acid hydrolysate of casein, and 0.15% starch.

#### 2.7.3. Application of Etest Strips

Etest^®®^ strips (HiMedia, India, Laboratories Pvt. Ltd., Haiderabad, India) for micafungin (0.002–32 µg/mL) and anidulafungin (0.002–32 µg/mL) were applied to the inoculated agar plates using sterile applicators. The plates were allowed to dry for 15 min at room temperature prior to incubation.

#### 2.7.4. Determination of Minimal Inhibitory Concentrations (MICs) and Susceptibility

MIC values were recorded at 24 and 48 h, defined as the lowest concentration at which the inhibition ellipse intersected the strip. MIC_50_ and MIC_90_ values, representing the MICs inhibiting 50% and 90% of isolates, respectively, were calculated to summarize overall susceptibility and detect potential resistance trends.

#### 2.7.5. Quality Control

Quality control of the minimum inhibitory concentration (MIC) assay for anidulafungin and micafungin was performed using reference strains *C. parapsilosis* ATCC 22019 and *Pichia kudriavzevii* ATCC 6258. MIC values against these strains fell within the acceptable ranges specified by HiMedia guidelines, confirming the accuracy and reliability of the assay.

## 3. Results

### 3.1. pH Range for Growth

pH is an important factor affecting the growth and vitality of all living organisms. *N. glabratus* is known for its adaptability. The experiments carried out showed growth in wide ranges of pH, namely from 3–9. Cultivation was performed out aerobically at 25 °C for 5 days in the basal YPD. The sample profiles were similar with minor variations.

### 3.2. Enzyme Secretion

#### 3.2.1. Enzyme Activities—Lipase, Esterase, Protease, Amilase, Β-Glucosidase

Secretion of some hydrolytic enzymes is a significant determinant of pathogenicity in *N. glabratus*. In addition, lipases and esterases are attributed an important role in the pathogen–host interaction. In light of this statement, the expectations for positive lipase and esterase activity in all clinical isolates, including the wild strain, were confirmed (Table 1).

All 48 strains exhibited positive lipase activity (Table 1). In most cases, the diameter of the clear zones ranged from 1.0 to 1.3 cm. The smallest zone was recorded for strain P13 (0.7 cm), while isolates P5, P6, P7, P19, P68, P73, and P95 displayed zones of 0.8–0.9 cm.

*Nakaseomyces glabratus* strains were also tested for esterase activity. After 120 h of incubation, the strains produced precipitated zones ranging from 0.9 to 1.5 cm around the colonies. Notably, six strains (P11, P12, P16, P24, P44, P93) exhibited weaker esterase activity with a zone diameter of 0.9 cm, while two strains (P20 and P71) showed markedly higher activity with a 1.5 cm zone.

The observed variability in enzymatic activity among the isolates may reflect metabolic adaptations to different food environments, strain-specific characteristics, or the origin of the clinical sample.

None of the tested isolates exhibited proteolytic or amylolytic activity.

#### 3.2.2. Assimilation of Arbutin

Arbutin is well known for its significant antiseptic, anti-inflammatory, and antimicrobial properties, and it is widely incorporated into therapeutic preparations for the treatment of urinary and gastrointestinal disorders. It can be enzymatically hydrolyzed by β-glucosidase, yielding hydroquinone and glucose, with hydroquinone recognized as the principal bioactive compound responsible for its antimicrobial effects [43]. In the present study, none of the clinical or wild-type *N*. *glabrata* isolates demonstrated the ability to assimilate arbutin.

### 3.3. Biofilm Formation

Biofilm formation plays a crucial role in fungal pathogenesis. In the present study, all clinical isolates as well as the wild-type isolate demonstrated the ability to form biofilms. In contrast to enzymatic activities such as lipase and esterase production, which showed variability, biofilm formation was consistent across all isolates. The measured optical density values at 595 nm ranged from 3.3 to 3.6.

#### EPS Synthesis

The ability of yeasts to synthesize EPS in a selective medium was explored. Cultivation was performed in 100 mL shaking flasks consisting of 25 mL of culture medium at 37 °C for 48 h on a rotary shaker at 220 rpm in a minimal salt medium with carbon source sucrose—4%, where the pH of the medium before sterilization was 4.3 and the medium was 37 °C. No significant differences were observed in the quantities of synthesized biopolymer, which varied within the range of 450–500 µg/mL.

### 3.4. Antifungal Activity

No significant growth inhibition of *C. parapsilosis* by *N. glabratus* was observed under the tested conditions. This suggests that in vitro, *N. glabratus* does not exert competitive suppression against *C. parapsilosis*, at least through direct growth inhibition mechanisms.

### 3.5. Antifungal Drug Resistance

According to the most recent guidelines issued by the European Committee on Antimicrobial Susceptibility Testing (EUCAST, version 11.0, 2024; (https://www.eucast.org/ast_of_fungi; last accessed on 20 May 2025)), *N. glabratus* isolates are categorized as susceptible (S) to micafungin when MIC values are ≤0.03 µg/mL and resistant (R) when MIC values exceed 0.03 µg/mL. For anidulafungin, susceptibility is defined by MIC values ≤ 0.06 µg/mL, while resistance is indicated by MIC values > 0.06 µg/mL.

Table 2 presents the distribution of 48 *N. glabratus* isolates classified as susceptible or resistant to micafungin and anidulafungin according to the EUCAST breakpoints, including MIC ranges, MIC₅₀, and MIC₉₀ values. MIC₅₀ and MIC₉₀ represent the MIC values inhibiting 50% and 90% of isolates, respectively, providing a summary of the overall susceptibility profile. As it can be seen, none of the 48 isolates were classified as susceptible to micafungin, exhibiting minimum inhibitory concentration (MIC) values ranging from 0.125 to 16 µg/mL, corresponding to 100% resistance. The MIC₅₀ and MIC₉₀ values for micafungin were 0.19 µg/mL and 8 µg/mL, respectively, indicating a broad distribution of reduced susceptibility among the isolates. Conversely, 27 (56%) of isolates were susceptible to anidulafungin, with MIC values spanning 0.004 to 16 µg/mL. The MIC₅₀ and MIC₉₀ values for anidulafungin were 0.032 µg/mL and 3 µg/mL, respectively. Resistance to both micafungin and anidulafungin was exhibited by 21 isolates (44%).

## 4. Discussion

This study investigated the biochemical and physiological profiles of forty-seven clinical isolates and one environmental isolate of *N. glabratus*. The research focused on key properties associated with *N. glabratus* pathogenicity while also evaluating traits not typically characteristic of this species, thereby providing insights into its biological variability.

The occurrence of *N. glabratus* in diverse ecological niches suggests a pronounced capacity for environmental adaptation. Effective pH tolerance is particularly critical for survival at different host sites, influencing virulence expression [11]. Wu et al. (2015) [44] demonstrated via proteomic analyses that low pH environments are less stressful for *N. glabratus* than high pH, a finding corroborated by our data. In the present study, isolates exhibited growth across a pH range of 3.0 to 9.0. Conversely, Huang et al. (2019) [45] reported growth inhibition at a lower pH due to acid accumulation. Environmental factors such as host absence, immune suppression, and competitive microbial populations strongly influence the behavior of *N. glabratus*, as they do for most opportunistic pathogens.

Hydrolytic enzyme secretion represents a major virulence factor in *N. glabratus*. In this study, strain P13 exhibited the smallest enzymatic activity zone (0.7 cm), while isolates P5, P6, P7, P19, P68, P73, and P95 showed halos ranging from 0.8 to 0.9 cm. The remaining isolates produced clear zones between 1.0 and 1.3 cm. Esterase activity was detected in all strains, with isolates P20 and P71 demonstrating the highest activity levels. Notably, negative esterase activity on Tween 80 has been reported previously in oral isolates of *N. glabratus* [46]. The variability in esterase and lipase activities likely reflects metabolic adaptations to environmental conditions, strain-specific traits, and inherent variability— hallmarks of opportunistic yeast infections. Differences in enzymatic profiles may also be influenced by clinical sample source, patient age, and underlying comorbidities.

Protease activity, which facilitates fungal survival across a broad pH range (2.0–7.0), was absent in all isolates, including the environmental strain. Rasheed et al. (2019) [47] attributed this to the predominance of glycosylphosphatidylinositol-anchored aspartyl proteases (CgYps1–11) in *N. glabratus*, which are essential for intracellular survival and virulence [48]. This finding aligns with our observations.

To further explore metabolic capabilities beyond pathogenicity-associated enzymes, we examined polysaccharide assimilation, focusing on soluble starch. None of the isolates exhibited amylolytic activity, consistent with prior reports, indicating the absence of amylase synthesis in *N. glabratus* [49]. While variability was evident in lipase and esterase activities, protease and amylase profiles remained uniformly negative across isolates.

Arbutin, an antimicrobial compound hydrolyzed by β-glucosidase in the gut, exerts antiseptic effects under alkaline conditions [50]. In this study, neither clinical nor environmental *N. glabratus* isolates assimilated arbutin, suggesting a potential therapeutic option targeting this metabolic deficiency in resistant strains.

Biofilm formation is regarded as the most concerning virulence trait of *N. glabratus* [51,52], contributing to treatment failures via enhanced antimicrobial tolerance. In our study, all isolates formed biofilms, corroborating existing data and partially explaining the global increase in yeast infections. Although little is known about the physiological changes accompanying biofilm development, proteomic profiling of *N. glabratus* biofilms has been reported [53]. Interestingly, cases of isolates lacking biofilm formation, protease, and esterase activity have been described, though these are rare among clinical isolates of opportunistic yeasts [54]. Biofilm formation is closely linked to exopolymer synthesis, which facilitates host adhesion—a key mechanism by which *Candida* species evade host defenses [55]. Among extracellular biofilm components, β-1,3-glucan is particularly important in protecting biofilms from antifungal agents [56]. In our batch culture experiments, EPS synthesis reached 450–500 µg/mL after 48 h, with minor inter-strain variation.

Data on the interaction or competitive behavior between non-*Candida* albicans complex (NCAC) species remain limited. We investigated potential antagonism between *N. glabratus* and *C. parapsilosis*. Despite *N. glabratus* being typically more invasive and adaptable, no competitive inhibition against *C. parapsilosis* was observed in vitro. This suggests that *N. glabratus* does not exert direct growth-suppressive effects under the tested conditions.

A key aspect of this study was antifungal susceptibility profiling and its relationship with enzymatic activities and biofilm formation. *N. glabratus* is increasingly recognized for its multidrug-resistant phenotype [57,58]. In our investigation, all isolates exhibited micafungin MICs above 0.03 µg/mL, and 21 isolates showed anidulafungin MICs exceeding 0.06 µg/mL. According to current EUCAST breakpoints, these isolates display multidrug resistance, closely associated with biofilm formation. It is widely acknowledged that biofilms impede antifungal penetration by establishing a diffusion barrier that binds charged antimicrobial molecules [51]. Echinocandin resistance in *N. glabratus* may result from acquired FKS mutations [59] or adaptive stress responses, leading to elevated cell wall chitin content and paradoxical growth in vitro [60]. Notably, while anidulafungin and micafungin share a common mechanism of action, 25% (12/48) of isolates remained susceptible to anidulafungin, demonstrating intra-class susceptibility variability—a phenomenon previously reported and attributed to differing affinities for mutant glucan synthase complexes [61,62]. These findings underscore the necessity for individual MIC determination for each echinocandin when treating *N. glabratus* infections rather than relying on class-wide assumptions. Furthermore, routine molecular screening for FKS mutations is recommended to enhance the detection of echinocandin resistance and guide antifungal therapy, particularly in invasive candidiasis cases [63]. The persistently high resistance rates observed in this study emphasize the urgent need for ongoing surveillance and antifungal stewardship, further reinforced by the fact that the wild strain exhibited a similar profile to clinical isolates.

## 5. Conclusions

This study establishes valuable phenotypic data on *N*. *glabratus* populations isolated in Bulgaria, addressing a significant gap in regional epidemiological knowledge. The demonstrated variability in enzymatic activity, consistent biofilm formation, and notable resistance to echinocandins confirm the adaptive potential and clinical relevance of this opportunistic pathogen. The absence of competitive inhibition against *C. parapsilosis* further underscores its distinct ecological behavior. The high resistance rates to micafungin and anidulafungin highlight the need for continuous surveillance and optimized antifungal use to maintain therapeutic efficacy. Collectively, these findings reinforce *N. glabratus* as an emerging public health concern and emphasize the necessity for continued regional surveillance and the development of the targeted antifungal management strategies.

## Figures and Tables

**Table 1:** Profile of enzymatic activities of 47 clinical isolates and environmental isolate D 77_3.

Strain №	Lipase Activity for 5 Days, Clear Zone, cm	Esterase Activity for 5 Days, Precipitated Zone, cm	Esterase Activity for 7 Days, Precipitated Zone, cm	Protease Activity for 5 Days	Amilase Activity for 5 Days	Β-Glucosidase Activity for 10 Days
P1	1.3	1.0	unchanged	-	-	-
P2	1.1	1.3	unchanged	-	-	-
P3	1.1	1.4	unchanged	-	-	-
P4	1.3	1.4	unchanged	-	-	-
P5	0.9	1.3	unchanged	-	-	-
P6	0.8	1.1	unchanged	-	-	-
P7	0.8	1.2	unchanged	-	-	-
P8	1.1	1.2	unchanged	-	-	-
P9	1.0	1.0	unchanged	-	-	-
P10	1.2	1.0	unchanged	-	-	-
P11	1.2	0.9	unchanged	-	-	-
P12	1.2	0.9	unchanged	-	-	-
P13	0.7	1.1	unchanged	-	-	-
P14	1.0	1.2	unchanged	-	-	-
P15	1.2	1.2	unchanged	-	-	-
P16	1.1	0.9	unchanged	-	-	-
P17	1.3	1.2	unchanged	-	-	-
P18	1.0	1.4	unchanged	-	-	-
P19	0.8	1.0	unchanged	-	-	-
P20	1.1	1.0	1.5	-	-	-
P24	1.3	0.9	unchanged	-	-	-
P26	1.2	1.3	unchanged	-	-	-
P28	1.2	1.0	unchanged	-	-	-
P29	1.2	1.3	unchanged	-	-	-
P32	1.1	1.4	unchanged	-	-	-
P37	1.3	1.4	unchanged	-	-	-
P38	1.3	1.4	unchanged	-	-	-
P39	1.1	1.0	unchanged	-	-	-
P41	1.1	1.2	unchanged	-	-	-
P44	1.3	0.9	unchanged	-	-	-
P68	0.9	1.0	unchanged	-	-	
P70	1.2	1.2	unchanged	-	-	-
P71	1.1	0.9	1.5	-	-	-
P73	0.8	1.3	unchanged	-	-	-
P75	1.2	1.4	unchanged	-	-	-
P79	1.3	1.0	unchanged	-	-	-
P87	1.3	1.0	unchanged	-	-	-
P90	1.1	1.1	unchanged	-	-	-
P92	1.1	1.0	unchanged	-	-	-
P93	1.3	0.9	unchanged	-	-	-
P95	0.9	1.2	unchanged	-	-	-
P97	1.2	1.4	unchanged	-	-	-
P99	1.3	1.0	unchanged	-	-	-
P100	1.1	1.1	unchanged	-	-	-
P102	1.3	1.3	unchanged	-	-	-
P105	1.2	1.2	unchanged	-	-	-
P106	1.2	1.0	unchanged	-	-	-
D77_3 wild strain	1.2	1.0	unchanged	-	-	-

**Table 2:** MIC ranges of micafungin and anidulafungin, and MIC₅₀ and MIC₉₀ values (MICs inhibiting 50% and 90% of isolates). The number and percentage of *N. glabratus* isolates classified as susceptible or resistant. Susceptibility is defined as MIC ≤ 0.03 µg/mL for micafungin and ≤0.06 µg/mL for anidulafungin, based on EUCAST v11.0 (2024) breakpoints.

Antifungal	MIC Range (μg/ml)	MIC_50_ (μg/ml)	MIC_90_ (μg/ml)	Susceptible, n	Resistantn, n
Micafungin	0.125–16	0.19	8	0 (0%)	48 (100%)
Anidulafungin	0.004–16	0.032	3	27 (56%)	21 (44%)

## Data Availability

The antifungal susceptibility profiles of the yeast isolates are available from the corresponding author upon reasonable request. The sequences generated in this study have been submitted to the GenBank database under the accession numbers ON016539–ON016585 for clinical isolates and JQ026352.1 for isolate D77_3.
